# Q-value customized versus wavefront-optimized ablation in femtosecond laser-assisted LASIK for myopia and myopic astigmatism: a prospective contralateral comparative study

**DOI:** 10.1186/s40662-022-00312-3

**Published:** 2022-11-02

**Authors:** Magdi Mohammad Mostafa, Hazem Abdelmotaal, Khaled Abdelazeem, Islam Goda, Mahmoud Abdel-Radi

**Affiliations:** Department of Ophthalmology, Assiut University Hospital, Assiut University, Sixth Floor, Assiut, 71516 Egypt

**Keywords:** Custom Q, Wavefront-optimized, Corneal asphericity, Q-value

## Abstract

**Background:**

Corneal refractive surgery for myopia results in an oblate shift with increased postoperative aberrations inversely affecting the quality of vision. Aspheric ablation profiles have been introduced to minimize such a problem. The aim of this study was to compare changes in corneal asphericity, central and mid-peripheral pachymetry between the Q-value customized and the wavefront-optimized (WFO) ablation profiles.

**Methods:**

A prospective, comparative non-randomized fellow eye study was conducted. Eighty eyes of 40 eligible patients underwent femtosecond laser-assisted laser in situ keratomileusis for myopia and myopic astigmatism. In each patient, the more myopic eye was included in the custom-Q ablation experimental group and the other less myopic eye was included in the WFO control group. For the custom-Q group, the target asphericity was set to the preoperative Q-value. Corneal asphericity, central and mid-peripheral pachymetric changes and the root mean square of corneal higher-order aberrations (RMSh) were assessed 6 months following surgery. Visual and refractive outcomes were also evaluated in both platforms 6 months postoperatively.

**Results:**

The mean preoperative refractive spherical equivalent was significantly more myopic in the custom-Q group than in the WFO group (*P* = 0.001). The mean Q-value changed from − 0.2 ± 0.1 to 0.6 ± 0.7 and from − 0.2 ± 0.1 to 0.4 ± 0.5 in the custom-Q and WFO groups, respectively. The oblate shift in corneal asphericity was not significantly different between both treatment groups (*P* = 0.094). The mean ablation depth at the pupillary center was significantly greater in the custom-Q group (*P* = 0.011), while there was no significant difference at the mid-peripheral pachymetry (*P* = 0.256). The RMSh significantly increased in both treatment profiles (*P* < 0.001) with no significant difference between the two groups (*P* = 0.06). The uncorrected distance visual acuity (UDVA) and the manifest refraction spherical equivalents (MRSE) significantly improved in both treatment groups (*P* < 0.001).

**Conclusions:**

The custom-Q treatment profile with target asphericity set at the preoperative Q-value achieved comparable outcomes *vs.* the WFO profile in terms of postoperative corneal asphericity and mid-peripheral pachymetry despite the greater amount of ablation, the smaller optical zone, and the resulting increase in postoperative corneal flattening in the custom-Q group.

*Trial registration* (Clinicaltrials.gov): NCT04738903, 4 February 2021- Retrospectively registered, https://clinicaltrials.gov/ct2/show/NCT04738903

## Background

Myopia and high myopia which are main causes of decreased unaided visual acuity are significantly increasing globally [1]. It is largely accepted as an epidemic and thus raises the need for measures to prevent its onset and slow down its progression as well as promoting the access to appropriate and accurate refractive correction [2–4].

Normally, the cornea is aspheric in shape and changes in the radius of the curvature from the apex to the periphery is expressed numerically as the “Q-value”. Curvature flattening or steepening towards the corneal periphery has a negative or a positive Q-value, respectively. In the general population, the Q-values range from − 0.8 to + 0.4 with a mean of − 0.2 [5, 6]. The average Q-value associated with the least spherical aberrations was found to be about − 0.27 on corneal topography and about − 0.53 on whole eye wavefront aberrometry [7–10]. A midway value of about − 0.4 was suggested based on an aspheric eye model [11].

Unfortunately, the standard treatment of myopia with the conventional laser assisted in situ keratomileusis (LASIK) is inevitably associated with central corneal flattening and change in asphericity towards the oblate shape (increase in Q-value). This oblate shift is associated with the development of higher-order aberrations (HOAs) and degradation of the quality of vision, in terms of contrast sensitivity and night vision, despite the improvement in visual acuity [12, 13]. Many algorithms were introduced to tackle this problem, the so-called “aspheric ablation profiles”, such as the wavefront-guided (WFG), wavefront-optimized (WFO) and the custom-Q factor ablation profiles [14].

The WFO ablation profile was designed to pre-compensate for the expected amount of HOAs induced by the conventional LASIK in the average eye but without changing the pre-existing optical aberrations. The peripherally sloping cornea leads to obliquity of the angle of incidence of the conventional LASIK pulses, known as the cosine effect [15], which leads to a significant decrease in the efficacy of ablation and hence a decrease in the ablation depth intended in the cornea’s periphery. The WFO profile in myopic correction significantly increases the depth of ablation in the mid-periphery of the optical zone by up to 35% than in the classic profile. It helps preserving the normal corneal prolateness but doesn't aim for adjusting the amount of corneal asphericity [6, 14].

The aspheric ablation profile of Q-adjusted treatments offers the ability to obtain a desired postoperative asphericity target, through modifying the mean corneal asphericity by symmetrically adjusting the number of laser pulses in the mid-periphery of the optical zone [16, 17]. However, the Q-adjusted treatments may lead to hypo- or hypercorrections derived from changes in corneal asphericity, especially with higher errors, necessitating adjustments in the refractive nomogram [18–20].

Previous studies compared different aspheric ablation profiles with special reference to the observed changes in central pachymetry or pachy-apex using variably adjusted custom-Q ablation nomograms [21–23]. The aim of this study is to compare the Q-value customized (asphericity-guided) and WFO ablation profiles focusing on the observed changes in mid-peripheral pachymetry. We also investigated the keratorefractive outcomes of the custom-Q ablation platform utilizing the preoperative Q-value as the target asphericity to better compare the impact of both ablation profiles on corneal asphericity as well as to decrease the need for custom-Q ablation nomograms suggested by earlier studies [19, 24].

## Patients and methods

### Study design and patient selection

This prospective, comparative non-randomized fellow eye study was conducted at Tiba Eye Center (private practice) from June 2020 to June 2021 and included 80 eyes of 40 consecutive patients. All patients were informed about the risks and benefits of the procedure and a written informed consent was obtained. The study included candidates undergoing femtosecond laser-assisted laser in situ keratomileusis (FS-LASIK) for myopic correction with or without myopic astigmatism. Our inclusion criteria were eyes with manifest refraction spherical equivalent (MRSE) up to − 12 diopters (D), corneal thickness at thinnest location ≥ 500 μm with the estimated postoperative residual stromal bed (RSB) of at least 60% of the thinnest pachymetry [25].

We excluded patients with unilateral myopia, hyperopic refractive error, mixed astigmatism, previous corneal surgery, ocular diseases, and those with contraindications to keratorefractive surgery such as significant dry eye, recurrent epithelial erosions, systemic autoimmune diseases and pregnant or lactating females.

In a fellow-eye study pattern, the eye that had a greater myopic MRSE was included in the custom-Q treatment group (experimental group) while the other eye with the less myopic MRSE was included in the WFO treatment group (control group). Eyes in each group were further stratified according to the degree of myopia into mild myopia (MRSE up to − 3.00 D), moderate myopia (MRSE from − 3.00 D to − 6.00 D), and high myopia (MRSE higher than − 6.00 D).

### Preoperative assessment

Preoperative assessment included a detailed history taking, exclusion of contact lens use in the past two weeks and a complete ocular examination. Refractive assessment involves measurement of the uncorrected distance visual acuity (UDVA), manifest refraction, corrected distance visual acuity (CDVA) using logarithms of minimum angle of resolution (logMAR) 4 m chart (Sussex Vision, Inc., Rustington, UK), and cycloplegic refraction. Slit-lamp biomicroscopy of anterior and posterior segment, intraocular pressure (IOP) assessment and Schirmer I test were also evaluated. Corneal tomography using the Pentacam (Oculus Pentacam HR, Oculus GmbH, Germany) was the standard tool for keratorefractive evaluation. The Pentacam capture was automatically triggered and only captures with good quality specification were used. Captures of low quality such as data gaps, blink, model or had extrapolated data to the central 8 mm were omitted. The preoperative Q-value for each eye was determined by calculating the average of three Q-value readings of three good quality captures (front surface Q-value at 6 mm). The root mean square of corneal HOAs (RMSh) was extracted from Pentacam by exporting data from OcuLink software to the EX500^®^ (WaveLight, Alcon lab, TX, USA) and then obtained from the ablation profile through the Zernike icon. The mean mid-periphery pachymetry was calculated for each eye by dividing the sum of 8 pachymetry points at the 4–6 mm zone in the pachymetry map by their numbers. One ophthalmologist (MM) performed all the preoperative clinical and investigatory assessments.

### Surgical technique

In both treatment groups, data entry in the EX500® treatment planning section included the patient’s refraction, K readings, thinnest pachymetry and optical zone diameter. In addition, the custom-Q profile data involved entry of Q1 and Q2 values (Q-values at the horizontal and vertical meridians in the topometric map of the corneal front at 6 mm, the average of both values is the front surface Q-value at 6 mm). The target postoperative Q-value was set the same value of the preoperative Q-value.

The target postoperative refraction was set to emmetropia. The only exception for intentional refractive undercorrection was if the assumed RSB was out of the safe thickness limits even with decreasing the optical zone to 6 mm. The Wellington nomogram was the nomogram of choice implemented for both groups as recommended by the manufacturer regardless of the degree of myopia or optical zone diameter.

All patients underwent corneal flap creation using the femtosecond laser WaveLight FS200^®^ (Alcon lab, TX, USA). The FS200 used a 200 kHz repetition rate, 1030 nm wavelength, a 6 µm spot size and laser pulse energy set at 0.8 µJ. The flap diameter was planned to be 9 mm in diameter with a superior hinge and a side cut angle of 90° in all eyes, while flap thickness ranged from 90 to 130 μm according to the patient’s keratorefractive profile and the surgeon’s preference. Excimer laser for myopia and/or myopic astigmatism correction was then performed using WaveLight EX500^®^. Postoperative treatment included a combination of Dexamethasone 0.1% and Tobramycin 0.3% eye drops four times daily for one week (TobraDex, Alcon) and topical lubricant eye drops containing Propylene Glycol 0.3% and Polyethylene Glycol-400 0.4% four times daily for 3 months (Systane Ultra, Alcon). One surgeon (MA) performed all FS-LASIK surgeries.

### Postoperative follow-up

Follow-up visits were scheduled at the first postoperative day, 2 weeks, 1 month, and 6 months postoperatively. Masking of the outcome assessor was applied at each follow-up visit. One of the authors (who was not involved in the non-random contralateral eye selection, preoperative assessment, and surgical intervention) performed the postoperative clinical and investigatory assessment using the same preoperative tools being masked from knowing the aspheric ablation platform used. At each visit, slit-lamp examination, UDVA, CDVA, residual refractive error with calculation of postoperative MRSE were documented. Pentacam was scheduled 6 months following surgery to assess postoperative topography, front surface Q-value at 6 mm, RMSh, K readings, central and mid-peripheral pachymetry. The actual depth of stromal tissue ablated was calculated by subtracting the postoperative pachymetry value from the preoperative pachymetry value.

### Statistical analyses

Statistical analyses were performed using the Statistical Package for Social Science software for Windows version 20.0 (Armonk, NY: IBM Corp, Inc, USA). The normal distribution of data was tested using the Shapiro-Wilk’s test. Descriptive statistics were evaluated to compare patients’ characteristics between groups. Independent samples t-test or the Mann-Whitney U test was used to compare the differences between the two treatment groups when the data were normally or non-normally distributed, respectively. On the other hand, the paired samples t-test or the Wilcoxon signed-rank test was used to compare the preoperative and postoperative values within each treatment group when the data were normally or non-normally distributed, respectively. Spearman’s rank-order correlation coefficient was used to measure the strength and direction of association between variables. *P* values less than 0.05 were considered statistically significant.

The sample size was calculated using G power software v.3.1.3 utilizing t-test for comparison of differences between two means assuming effect size of 0.28. Alpha error probability was set at 0.05 and power of 0.8 was used.

## Results

This contralateral eye study comprised 80 eyes of 40 patients, 16 males (40%) and 24 females (60%), with a mean age of 28.12 ± 7.55 years (range, 20 to 47 years). All patients completed their 6-month follow-up schedule.

### Preoperative data

The preoperative values of the spherical error and MRSE were significantly higher in the custom-Q group than the WFO group, while the UDVA and CDVA were significantly lower. Other parameters showed no significant preoperative differences between the two treatment groups (Table 1). On further analysis of the refractive error into mild, moderate, and high myopia, there were significantly more myopic spherical errors and MRSEs (*P* = 0.014 and *P* = 0.019, respectively) in the custom-Q than the WFO group in the high myopia subgroup, but non-significant differences were observed in the mild and moderate myopia subgroups.

**Table 1:** Preoperative data of both treatment groups

Parameter	Ablation profile groups Mean ± SE 95% CI (LB, UB)		*P* value
	Custom-Q profile (n = 40)	WFO profile (n = 40)	
UDVA (logMAR)	1.30 ± 0.03 (1.23, 1.36)	1.10 ± 0.05 (1.00, 1.20)	**0.009**
CDVA (logMAR)	0.28 ± 0.03 (0.22, 0.35)	0.17 ± 0.03 (0.10, 0.20)	**0.010**
Sphere (D)	− 6.20 ± 0.48 (− 7.15, − 5.23)	− 4.10 ± 0.40 (− 4.90, − 3.30)	**0.001**
Cylinder (D)	− 1.70 ± 0.20 (− 2.10, − 1.30)	− 1.50 ± 0.20 (− 1.90, − 1.10)	0.489
MRSE (D)	− 7.00 ± 0.48 (− 8.00, − 6.10)	− 4.90 ± 0.40 (− 5.70, − 4.0)	**0.001**
K_1_ (D)	43.30 ± 0.24 (42.80, 43.80)	43.30 ± 0.30 (42.80, 43.90)	0.967
K_2_ (D)	44.80 ± 0.24 (44.30, 45.30)	44.60 ± 0.30 (44.10, 45.20)	0.582
K_m_ (D)	44.00 ± 0.24 (43.60, 44.50)	44.00 ± 0.30 (43.50, 44.50)	0.852
Q value	− 0.20 ± 0.02 (− 0.25, − 0.17)	− 0.20 ± 0.02 (− 0.25, − 0.16)	0.712
Pachy. pupil center (μm)	539.00 ± 4.50 (530, 548)	539.00 ± 4.50 (530, 548)	0.973
Mid-peripheral Pachy. (μm)	572. 00 ± 4.80 (562, 582)	574.00 ± 4.70 (564, 583)	0.744
Degree of myopia, eyes *n* (%)			
Mild (≤ − 3.00 D) Moderate (− 3.00 to − 6.00 D) High (≥ − 6.00 D)	6 (15.0%) 9 (22.5%) 25 (62.5%)	12 (30.0%) 15 (37.5%) 13 (32.5%)	

*SE* = standard error of the mean; *CI* = confidence interval; *LB* = lower bound; *UB* = upper bound; *UDVA* = uncorrected distance visual acuity; *CDVA* = corrected distance visual acuity; *logMAR* = logarithms of minimum angle of resolution; *MRSE* = manifest refraction spherical equivalent; *D* = diopter; *K*_*1*_ = flat keratometry; *K*_*2*_ = steep keratometry; *K*_*m*_ = mean keratometry; *Pachy.* = pachymetry; *WFO* = wavefront-optimized. *P* values in bold indicate statistical significance

### Visual outcome

The UDVA significantly improved, while the CDVA showed non-significant change in each of the treatment groups even through myopia subgrouping. However, both the UDVA and CDVA were significantly better in the WFO than in the custom-Q group in preoperative and postoperative follow-up (Table 2). In each of myopia subgroup, there were non-significant differences between the two treatment groups in UDVA and CDVA regarding the preoperative, postoperative and the amount of change.

**Table 2:** Visual and refractive outcomes in both treatment groups

Parameter		Ablation profile groups Mean ± SE 95% CI (LB, UB)		*P* value ^b^
		Custom-Q profile (n = 40)	WFO profile (n = 40)	
UDVA (logMAR)	Preoperative	1.30 ± 0.03 (1.23, 1.36)	1.10 ± 0.05 (1.00, 1.20)	**0.009**
	Postoperative	0.40 ± 0.04 (0.28, 0.46)	0.20 ± 0.04 (0.14, 0.30)	**0.007**
	Difference	0.90 ± 0.04 (0.86, 1.00)	0.90 ± 0.04 (0.84, 1.00)	0.819
	*P* value^a^	**< 0.001**	**< 0.001**	
CDVA (logMAR)	Preoperative	0.28 ± 0.03 (0.22, 0.35)	0.17 ± 0.03 (0.10, 0.20)	**0.010**
	Postoperative	0.28 ± 0.03 (0.22, 0.35)	0.18 ± 0.03 (0.10, 0.20)	**0.014**
	Difference	0.000 ± 0.006 (− 0.010, 0.010)	− 0.008 ± 0.004 (− 0.020, 0.001)	0.417
	*P* value^a^	1.000	0.083	
Optical zone		6.20 ± 0.04 (6.16, 6.32)	6.40 ± 0.03 (6.32, 6.46)	**0.006**
Sphere (D)	Preoperative	− 6.20 ± 0.48 (− 7.15, − 5.23)	− 4.10 ± 0.40 (− 4.90, − 3.30)	**0.001**
	Postoperative	− 0.50 ± 0.09 (− 0.60, − 0.30)	− 0.10 ± 0.05 (− 0.20, 0.01)	**0.001**
	Difference	5.70 ± 0.40 (4.90, 6.60)	4.00 ± 0.40 (3.20, 4.80)	**0.003**
	*P* value^a^	**< 0.001**	**< 0.001**	
Cylinder (D)	Preoperative	− 1.70 ± 0.20 (− 2.10, − 1.30)	− 1.50 ± 0.20 (− 1.90, − 1.10)	0.489
	Postoperative	− 0.30 ± 0.07 (− 0.45, − 0.18)	− 0.20 ± 0.05 (− 0.30, − 0.10)	0.274
	Difference	1.40 ± 0.19 (0.98, 1.70)	1.30 ± 0.17 (0.90, 1.60)	0.892
	*P* value^a^	**< 0.001**	**< 0.001**	
MRSE (D)	Preoperative	− 7.00 ± 0.48 (− 8.00, − 6.10)	− 4.90 ± 0.40 (− 5.70, − 4.00)	**0.001**
	Postoperative	− 0.60 ± 0.10 (− 0.80, − 0.40)	− 0.20 ± 0.06 (− 0.32, − 0.08)	**0.001**
	Difference	6.40 ± 0.40 (5.60, 7.20)	4.70 ± 0.40 (3.90, 5.40)	**0.002**
	*P* value^a^	**< 0.001**	**< 0.001**	

*UDVA* = uncorrected distance visual acuity; *CDVA* = corrected distance visual acuity; *logMAR* = logarithms of minimum angle of resolution; *MRSE* = manifest refraction spherical equivalent; *WFO* = wavefront-optimized. *P* values in bold indicate statistical significance

^a^*P* value of change between pre- and postoperative values (intra-group)

^b^*P* value of difference between custom-Q and WFO groups (inter-group)

**Fig. 1:**
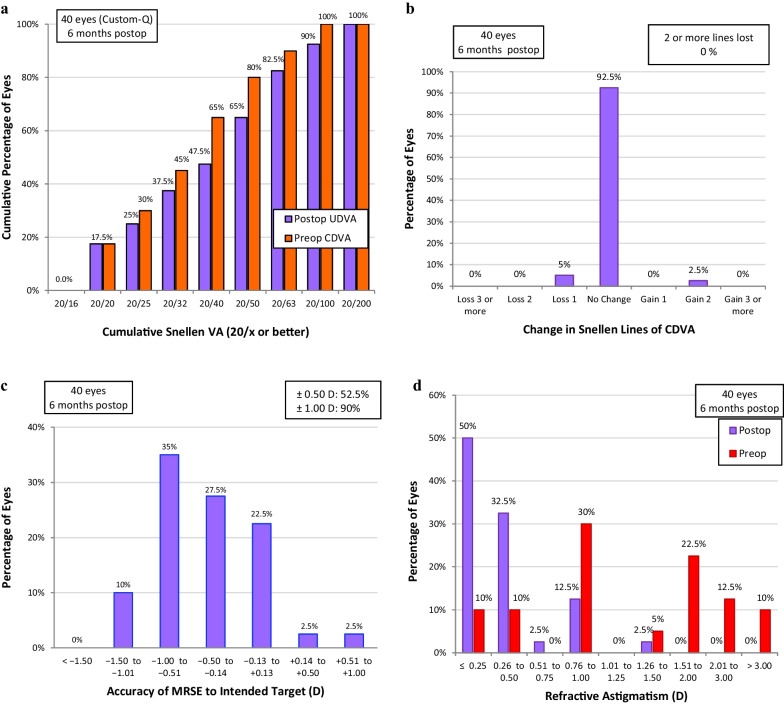
Visual and refractive outcomes in the custom-Q group 6 months postoperatively. **a** Cumulative postoperative Snellen UDVA as compared to the cumulative preoperative Snellen CDVA. **b** Difference between preoperative and postoperative CDVA in terms of lost or gained lines. **c** Proximity of the achieved to the intended spherical equivalent. **d** Preoperative and postoperative refractive cylinder. CDVA, corrected distance visual acuity; UDVA, uncorrected distance visual acuity

**Fig. 2:**
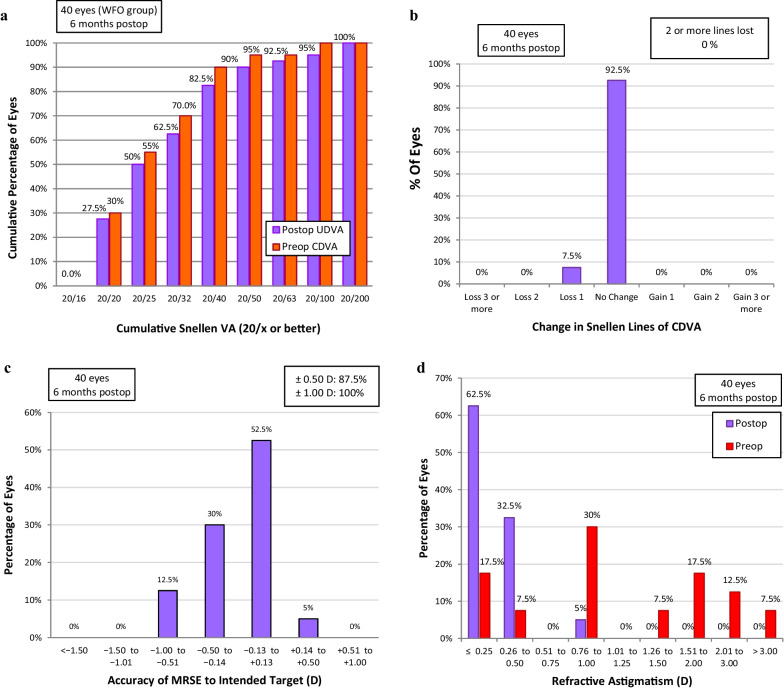
Visual and refractive outcomes in the wavefront-optimized group 6 months postoperatively. **a** Cumulative postoperative Snellen UDVA as compared to the cumulative preoperative Snellen CDVA. **b** Difference between preoperative and postoperative CDVA in terms of lost or gained lines. **c** Proximity of the achieved to the intended spherical equivalent. **d** Preoperative and postoperative refractive cylinder. CDVA, corrected distance visual acuity; UDVA, uncorrected distance visual acuity

Seven eyes in the custom-Q group (17.5%) achieved 20/20 UDVA (Fig. 1a); Four eyes in the mild myopia (66.7%) and three eyes in the moderate myopia subgroup (33.3%). For the WFO group, 11 eyes (27.5%) achieved 20/20 UDVA (Fig. 2a); Seven eyes in the mild myopia (58.3%) and four eyes in the moderate myopia subgroup (26.7%). None of the eyes achieved 20/20 in high myopia in either group. Collectively, for myopia up to − 6.00 D, 46.7% of custom-Q group and 40.7% of the WFO group achieved 20/20.

In the custom-Q group, one eye (2.5%) gained two lines and two eyes (5%) lost one line in the CDVA logMAR (in the high myopia subgroup), while 37 eyes (92.5%) had their preoperative CDVA preserved (Fig. 1b). The two eyes in the custom-Q group were in the high myopia subgroup and were treated using an optical zone of 6 mm; they had an oblate shift of 1.00 and 1.36, and an increase in the RMSh by 0.71 and 1.44 µm. In the WFO group, three eyes (7.5%) lost one line (two in the high myopia and one in the moderate myopia subgroup) while 37 eyes (92.5%) had their preoperative CDVA preserved (Fig. 2b). Two of the three eyes that lost one line in the WFO group were in the high myopia subgroup and were treated using an optical zone of 6 mm. They had an oblate shift of 1.12 and 0.30, and an increase in the RMSh by 0.93 and 0.42 µm, while the third eye had an oblate shift of 0.98 and an increase in the RMSh by 0.17 µm.

Seventeen eyes in the custom-Q group (42.5%) had anisometropia of ≥ 3.00 D (15 eyes in the high myopia subgroup and two eyes in the moderate myopia subgroup). The magnitude of anisometropia ranged from − 3.00 to − 6.25 D (mean ± SE: − 4.1 ± 0.3 D; 95% CI: − 3.50 to − 4.70 D). Out of these 17 eyes, there were 12 eyes (30% of the custom-Q group, 11 eyes in the high myopia subgroup and one eye in the moderate myopia subgroup) with suggested amblyopia as their preoperative CDVA were ≤ 20/32 (≥ 0.2 logMAR). They were worse than contralateral eyes of the WFO group by at least two lines with anisometropia of ≥ 3.00 D. The depth of amblyopia ranged from 0.2 to 0.5 logMAR lines, and it was two lines in five eyes, three lines in two eyes, four lines in four eyes and five lines in one eye. There was a weak and non-significant correlation between the magnitudes of anisometropia and the depths of amblyopia (Spearman coefficient r = 0.22, *P* = 0.499).

Spearman’s rank-order correlation was used to determine the relationship between the inter-ocular difference in MRSE (magnitude of anisometropia) and the difference in preoperative CDVA, which showed a strong positive correlation (r = 0.838, *P* < 0.001).

#### Refractive outcome and optical zone

Significant improvement of spherical and cylindrical errors and MRSE was noted in both treatment groups, but a significantly smaller optical zone had to be used in the custom-Q group (Table 2). The preoperative and postoperative MRSE were significantly more myopic, and the amount of error corrected was significantly greater in the custom-Q group (Table 2).

In the mild and moderate myopia subgroups, there was no significant difference in preoperative, postoperative or the amount of error corrected, between the custom-Q and the WFO groups. In the high myopia subgroup, the MRSE was significantly more myopic in the custom-Q group, both in the preoperative visit (*P* = 0.019) and postoperative visit (*P* = 0.009), while the amount of error corrected was not significantly different between the two treatment groups (*P* = 0.065).

In all eyes of the mild and moderate myopia subgroups, the optical zone was 6.5 mm. In the high myopia subgroup, the optical zone had to be decreased to 6 mm in 21 out of 25 eyes (84%) in the custom-Q group and in 9 out of 13 eyes (69%) in the WFO group. Even while decreasing the optical zone to 6 mm, six eyes in the high myopia subgroup of the custom-Q treatment group had to be undercorrected with intended postoperative MRSE of − 0.50 to − 0.75 D.

Fifteen eyes (37.5%) in the custom-Q group and 28 eyes (70%) in the WFO group were within 0.25 D of the intended target MRSE, and 21 eyes (52.5%) in the custom-Q group (Fig. 1c) and 35 eyes (87.5%) in the WFO group (Fig. 2) were within 0.50 D of the intended target MRSE. On analyzing the mild and moderate myopia subgroups together, 12 eyes (80%) in the custom-Q and 22 eyes (81.5%) in the WFO group were within 0.25 D of the intended target MRSE. In addition, 14 eyes (93.3%) in the custom-Q and 27 eyes (100%) in the WFO group were within 0.50 D of the intended target MRSE.

The cylindrical error showed statistically significant postoperative improvement in each treatment group, but non-significant difference was noted between the two groups in preoperative, postoperative and the amount of error corrected (Table 2, Figs. 1d and 2d).

#### Pachymetry change at pupillary center and mid-periphery

No significant difference between the two treatment groups was noted in the mean mid-peripheral pachymetry, while pachymetry at the pupillary center showed significantly thinner postoperative values in the custom-Q than WFO group (Table 3).

**Table 3:** Pachymetry changes in both treatment groups

Parameter	Ablation profile groups Mean ± SE 95% CI (LB, UB)		*P* value ^b^
	Custom-Q profile (n = 40)	WFO profile (n = 40)	
Pachy. pupil center (μm)			
Preoperative	539.00 ± 4.50 (530, 548)	539.00 ± 4.50 (530, 548)	0.973
Postoperative	449.00 ± 7.80 (433, 464.90)	470.00 ± 7.30 (455, 485)	**0.044**
Difference	90.00 ± 6.40 (77, 102.90)	69.00 ± 6.20 (57, 82)	**0.011**
* P* value^a^	**< 0.001**	**< 0.001**	
Mid-peripheral Pachy. (μm)			
Preoperative	572.00 ± 4.80 (562, 582)	574.00 ± 4.70 (564, 583)	0.744
Postoperative	519.00 ± 6.00 (506.70, 530.90)	527.00 ± 6.10 (514.40, 539.00)	0.397
Difference	53.00 ± 3.60 (45.90, 60.60)	46.9.00 ± 4.40 (38.00, 55.70)	0.256
* P* value ^a^	**< 0.001**	**< 0.001**	

*Pachy.* = pachymetry; *WFO* = wavefront-optimized. *P* values in bold indicate statistical significance

^a^*P* value of change between pre- and postoperative values (intra-group)

^b^*P* value of difference between custom-Q and WFO groups (inter-group)

The mean mid-peripheral pachymetry showed non-significant differences between custom-Q and WFO groups in all myopia subgroups for the preoperative, postoperative and amount of change. In mild myopia, the *P* values were 0.454, 0.888 and 0.512, respectively. In moderate myopia, the *P* values were 0.788, 0.270 and 0.152, respectively. In high myopia, the *P* values were 0.866, 0.723 and 0.890, respectively.

In the mild myopia subgroup, the mean postoperative mid-peripheral ablation depth was non-significantly greater in the custom-Q (23.8 ± 5.7) than the WFO group (18.5 ± 1.7). In the moderate and high myopia subgroups, the mean postoperative mid-peripheral ablation depth was non-significantly greater in the WFO group, 38.0 ± 5.8 for the custom-Q group and 51.5 ± 6.4 for the WFO group in the moderate myopia subgroup and 65.8 ± 3.1 for the custom-Q group and 67.7 ± 6.0 for the WFO group in the high myopia subgroup.

#### Keratometry readings

Both the custom-Q and the WFO ablation profiles led to a statistically significant flattening-effect on K_1_, K_2_ and K_m_ (*P* < 0.001 for each). The preoperative K_1_, K_2_ and K_m_ readings were non-significantly different in the two treatment groups, but the postoperative readings showed a statistically significantly greater flattening in the custom-Q treatment group (Table 4).

**Table 4:** Keratometry, Q-value and root mean square changes in both treatment groups

Parameter		Ablation profile groups Mean ± SE 95% CI (LB, UB)		*P* value^b^
		Custom-Q profile (n = 40)	WFO profile (n = 40)	
K_1_ (D)	Preop	43.30 ± 0.24 (42.80, 43.80)	43.30 ± 0.30 (42.80, 43.90)	0.967
	Postop	39.00 ± 0.44 (37.80, 39.60)	40.20 ± 0.40 (39.40, 40.90)	**0.014**
	Difference	4.60 ± 0.40 (3.80, 5.40)	3.20 ± 0.40 (2.40, 3.90)	**0.007**
	*P* value^a^	**< 0.001**	**< 0.001**	
K_2_ (D)	Preop	44.80 ± 0.24 (44.30, 45.30)	44.60 ± 0.30 (44.10, 45.20)	0.582
	Postop	39.20 ± 0.40 (38.40, 40.10)	40.60 ± 0.40 (39.80, 41.30)	**0.026**
	Difference	5.60 ± 0.40 (4.80, 6.40)	4.10 ± 0.40 (3.30, 4.80)	**0.003**
	*P* value ^a^	**< 0.001**	**< 0.001**	
K_m_ (D)	Preop	44.00 ± 0.24 (43.60, 44.50)	44.00 ± 0.30 (43.50, 44.50)	0.852
	Postop	39.00 ± 0.44 (38.00, 39.80)	40.40 ± 0.40 (39.60, 41.10)	**0.014**
	Difference	5.10 ± 0.40 (4.40, 5.90)	3.60 ± 0.40 (2.90, 4.40)	**0.003**
	*P* value^a^	**< 0.001**	**< 0.001**	
Q-value	Preop	− 0.20 ± 0.02 (− 0.25, − 0.17)	− 0.20 ± 0.02 (− 0.25, − 0.16)	0.712
	Postop	0.60 ± 0.11 (0.41, 0.84)	0.40 ± 0.08 (0.19, 0.53)	0.089
	Difference	0.80 ± 0.10 (0.63, 1.05)	0.60 ± 0.08 (0.40, 0.73)	0.094
	*P* value^a^	** < 0.001**	**< 0.001**	
RMSh (μm)	Preop	0.50 ± 0.03 (0.44, 0.55)	0.52 ± 0.04 (0.45, 0.60)	0.658
	Postop	1.00 ± 0.07 (0.86, 1.10)	0.83 ± 0.04 (0.74, 0.92)	0.133
	Difference	0.50 ± 0.06 (0.38, 0.63)	0.31 ± 0.04 (0.22, 0.40)	0.060
	*P* value^a^	**< 0.001**	**< 0.001**	

*K*_*1*_ = flat keratometry; *K*_*2*_ = steep keratometry; *K*_*m*_ = mean keratometry; *RMSh* = root mean square of corneal higher-order aberrations; *WFO* = wavefront-optimized. *P* values in bold indicate statistical significance

^a^*P* value of change between pre- and postoperative values (intra-group)

^b^*P* value of difference between custom-Q and WFO groups (inter-group)

#### Corneal asphericity

**Fig. 3:**
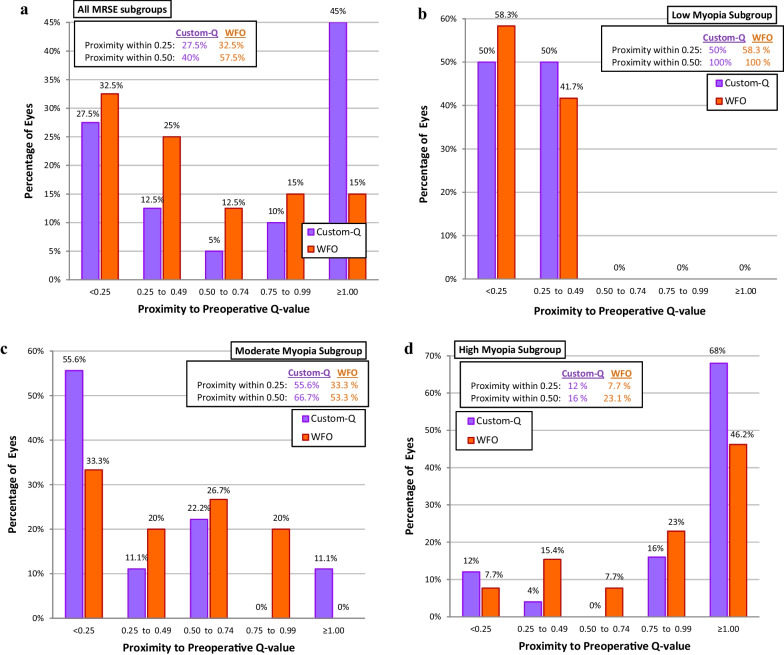
Proximity of the achieved to the preoperative Q-value in both treatment groups (taking the preoperative Q-value as the target asphericity for the custom-Q group). **a** All manifest refractive spherical equivalent (MRSE) subgroups; **b** Low myopia subgroup; **c** Moderate myopia subgroup; **d** High myopia subgroup

The Q-value showed a statistically significant oblate shift (*P* < 0.001) in each treatment group with non-significant difference between the mean postoperative Q-values of both groups (*P* = 0.089; Table 4, Fig. 3).

In the mild, moderate, and high myopia subgroups, both treatment profiles led to a statistically significant postoperative oblate shift, a *P* value of 0.027, 0.018 and less than 0.001, respectively in the custom-Q group, and a *P* value of 0.002, 0.001 and 0.002, respectively in the WFO group. However, on comparing both treatment profiles in each myopia subgroup, there were non-significant differences in preoperative, postoperative Q-value or the amount of oblate shift in mild myopia (*P* = 0.963, 0.925 and 0.605, respectively), in moderate myopia (*P* = 0.928, 0.233 and 0.270, respectively) and in the high myopia subgroup (*P* = 0.295, 0.415 and 0.242, respectively). The mean change in postoperative corneal asphericity showed statistically non-significant (*P* = 0.094) greater oblate shift in the custom-Q group (0.8 ± 0.1) than the WFO group (0.60 ± 0.08). On subgroup analysis, both treatment profiles had similar amounts of oblate shift in the mild myopia subgroup (0.20 ± 0.05 for the custom-Q and 0.20 ± 0.04 for the WFO group, *P* = 0.6). In the moderate myopia subgroup, the mean oblate shift was non-significantly greater in the WFO group than the custom-Q group (0.5 ± 0.1 and 0.3 ± 0.1, respectively, *P* = 0.27). In the high myopia group, the mean oblate shift was non-significantly greater in the custom-Q group than the WFO group (1.2 ± 0.1 and 1.0 ± 0.2, respectively, *P* = 0.24).

#### Root mean square of corneal HOAs (RMSh) at 6 mm zone

There was a statistically significant increase in the postoperative RMSh in both treatment groups (*P* < 0.001, Table 4). There was no significant difference between the two treatment groups regarding the preoperative (*P* = 0.658), postoperative (*P* = 0.133) and the amount of increase in the RMSh (*P* = 0.06).

The amount of increase in the RMSh, although non-significant, was greater in the custom-Q group than the WFO group (0.50 ± 0.06 and 0.31 ± 0.04 μm, respectively, *P* = 0.06). In the mild myopia subgroup, we found a non-significant greater increase in RMSh in the WFO than the custom-Q group (0.16 ± 0.02 and 0.10 ± 0.01 μm, respectively, *P* = 0.08). The RMSh also showed a non-significant greater increase in the WFO group than the custom-Q group (0.29 ± 0.06 and 0.17 ± 0.08 μm, respectively, *P* = 0.055) in the moderate myopia subgroup. In the high myopia subgroup, the custom-Q group had a non-significantly greater increase than the WFO group (0.73 ± 0.06 and 0.50 ± 0.10 μm, respectively, *P* = 0.069).

#### Subgroup analysis excluding eyes with amblyopia

A subgroup analysis was conducted and involved only eyes without amblyopia, where both treatment groups were compared. The results showed no significant difference in preoperative, postoperative and the amount of change in the UDVA, CDVA, optical zone, MRSE, pachymetry at pupillary center, mid-peripheral pachymetry, keratometry readings, corneal asphericity and RMSh. For corneal asphericity, the amount of oblate shift was almost equal in both groups as it was 0.6 ± 0.1 in the custom-Q and 0.60 ± 0.08 in WFO group with *P* = 0.901.

#### Correlations

A Spearman’s rank-order correlation was performed to assess the relationship between the preoperative MRSE and the postoperative Q-value in both custom-Q and WFO treatment groups. A strong, negative, statistically significant correlation in both treatment groups was found (r = − 0.74, r = − 0.6, respectively, *P* < 0.001). The same correlation was also noticed between the preoperative MRSE and the depth of ablation at the pupillary center and the mid-periphery in both treatment groups (r = − 0.8, *P* < 0.001). The amount of increase of RMSh showed a strong, negative, statistically significant correlation to the preoperative MRSE in the custom-Q group (r = − 0.87, *P* < 0.001), and in the WFO group (r = − 0.6, *P* < 0.001). Likewise, the optical zone showed a strong, negative, statistically significant correlation to the amount of increase in RMSh in the custom-Q group (r = − 0.74, *P* < 0.001), but there was a weak, negative, statistically significant correlation to RMSh change in the WFO group (r = − 0.3, *P* = 0.036).

## Discussion

Both custom-Q and WFO aspheric treatment platforms work through modifying the ablation profile at the mid-periphery of the optical zone from that of the conventional profile. Increasing the ablation depth at the mid-periphery to preserve prolateness is a strategy of the WFO profile while symmetrical adjustment of this increase in mid-periphery ablation is the strategy of the custom-Q profile [16, 17].

We compared the effect of each treatment profile on the mean mid-peripheral ablation depth and corneal asphericity when the target Q-value was set to the preoperative value in the custom-Q group. We found no significant difference between the custom-Q and the WFO treatment profiles in their effect on the mean mid-peripheral depth of ablation or corneal asphericity despite the greater amount of correction and the consequent greater flattening in the custom-Q group even when further studied in each myopia subgroup.

Unlike other studies [26, 27] that reported non-significant preoperative differences between the custom-Q and WFO groups, our study included eyes that were significantly more myopic with significantly lower visual acuities and detected amblyopia in 30% of eyes in the custom-Q group. Despite the significantly greater amount of correction in the custom-Q group, the postoperative MRSE remained significantly more myopic than in the WFO group, which can be partly explained by aiming for undercorrection in 15% of eyes in the custom-Q group. However, these significant differences were present only in the high myopia subgroup while there were no significant differences in mild and moderate myopia subgroups between the custom-Q and WFO eyes which was consistent with previous studies that included the same range of spherical equivalent [16, 26, 27].

Pachymetry at the pupillary center following surgery was significantly thinner in the custom-Q group than the WFO group. This was consistent with the significantly greater amount of myopic correction in the custom-Q group. Moreover, a significantly smaller optical zone had to be used to achieve such greater myopic correction in the custom-Q group without jeopardizing the safe RSB thickness. Previous reports did not report such differences between the two treatment groups [16, 26, 27]. Unlike the tendency of the central ablation towards a greater depth in custom-Q group, the mid-peripheral pachymetry ablation depth showed non-significant differences between the two treatment groups even with myopic subgroup stratification.

The flattening effect of myopic correction on keratometry readings was statistically significant in both treatment groups which is expected in any myopia correction protocol [17]. However, a significantly greater flattening effect in the custom-Q group was noted. This is consistent with the greater myopic correction in the custom-Q group and supported by previous reports that found a linear positive correlation between the degree of central flattening and the depth of ablation [17, 28].

In the current study, the target asphericity was set the same as the preoperative Q-value for eyes in the custom-Q group to compare the impact of both ablation profiles on corneal asphericity impartially. A statistically significant postoperative oblate shift was observed in all eyes in both treatment groups as demonstrated by prior studies [26–28] while there was no significant difference in the degree of oblate shift between the two groups. Our results showed a non-significantly higher postoperative oblate shift in the custom-Q group compared with the WFO group that can be explained by the greater refractive correction in addition to the significantly smaller optical zone in the custom-Q group. Smaller optical zones are associated with tendency towards a more oblate shift as suggested by Hou et al. [29]. On further subgroup analysis, a non-significant greater oblate shift was noted in the WFO group in the moderate myopia subgroup which is consistent with previous reports that dealt with a similar range of refractive error [16, 26]. On the other hand, a non-significant greater oblate shift was noted in the custom-Q group in the high myopia subgroup. Others reported that setting the target asphericity towards a more prolate target (around − 0.4) achieved a marginally significant lesser oblate shift in custom-Q group when compared with WFO [16] or WFG profile [17].

The root mean square of corneal higher-order aberrations (RMSh) were significantly different between the pre- and postoperative values in each treatment group, with non-significant differences on comparing the two treatment groups. Previous studies [30–32] supported our results that contradict Stojanovic et al.’s [16] study who reported that RMS of HOAs did not change significantly in both custom-Q and WFO eyes with moderate myopia who underwent surface ablation with a standard 6.5 mm optical zone diameter.

The postoperative corneal asphericity, the degree of oblate shift, the ablation depth at corneal mid-periphery and the amount of change in RMSh showed non-significant differences between the two treatment groups despite the significantly higher magnitude of corrected refractive error, greater central ablation depth, smaller optical zone and the more flattening effect in the custom-Q group.

Our study had a few limitations such as the lack of randomization with application of the custom-Q profile to the more myopic of the two eyes of each patient. Unexpectedly, the preoperative MRSE, anisometropia, CDVA and amblyopia were strongly significantly greater in the custom-Q group. This potential bias was partially solved by stratifying eyes into myopia subgroups. However, such a stratification had its own limitations in terms of unequal percentages of eyes in each myopia subgroup that may have resulted in a lower statistical power and potential statistical bias. On the other hand, finding out that the custom-Q profile has achieved comparable results to the WFO even when challenged with more myopic errors can be a worthwhile contribution. Other limitations include the lack of access to aberrometry and contrast sensitivity tools.

## Conclusions

The custom-Q treatment profile did not result in a more favorable outcome than the WFO ablation profile in terms of corneal asphericity and mid-peripheral depth of ablation. However, it should be taken into consideration that the custom-Q profile was challenged with a more myopic refractive error, a smaller optical zone, and consequently led to greater central ablation and corneal flattening in addition to using the preoperative Q-value instead of a more prolate target asphericity.

Further, software innovations are required to upgrade different aspheric ablation profiles to achieve an aberration-free vision with high level of patient satisfaction following refractive surgery.

## Data Availability

Data are available from the corresponding author upon reasonable request.

## Abbreviations

FS-LASIK: Femtosecond laser-assisted laser in situ keratomileusis
WFO: Wavefront-optimized
HOAs: Higher-order aberrations
WFG: Wavefront-guided
MRSE: Manifest refraction spherical equivalent
UDVA: Uncorrected distance visual acuity
CDVA: Corrected distance visual acuity
IOP: Intraocular pressure
LogMAR: Logarithm of the minimum angle of resolution
Km: Mean keratometry
RMSh: Root mean square of higher-order aberrations
RSB: Residual stromal bed

## References

[1] Holden BA, Fricke TR, Wilson DA, Jong M, Naidoo KS, Sankaridurg P (2016). Global prevalence of myopia and high myopia and temporal trends from 2000 through 2050. Ophthalmology.

[2] Cheung N, Lee SY, Wong TY (2021). Will the myopia epidemic lead to a retinal detachment epidemic in the future?. JAMA Ophthalmol.

[3] Cooper J, Tkatchenko AV (2018). A review of current concepts of the etiology and treatment of myopia. Eye Contact Lens.

[4] Németh J, Tapasztó B, Aclimandos WA, Kestelyn P, Jonas JB, De Faber JHN (2021). Update and guidance on management of myopia. European Society of Ophthalmology in cooperation with International Myopia Institute. Eur J Ophthalmol.

[5] Mrochen M, Donitzky C, Wüllner C, Löffler J (2004). Wavefront-optimized ablation profiles: theoretical background. J Cataract Refract Surg.

[6] Mrochen M, Lemanski N, Pajic B, Sinjab M, Cummings A (2018). Optical physics of customized laser ablation profiles. Customized laser vision correction.

[7] Holladay JT (2015). Effect of corneal asphericity and spherical aberration on intraocular lens power calculations. J Cataract Refract Surg.

[8] Beiko GH, Haigis W, Steinmueller A (2007). Distribution of corneal spherical aberration in a comprehensive ophthalmology practice and whether keratometry can predict aberration values. J Cataract Refract Surg.

[9] Sinjab MM, Sinjab M, Cummings A (2018). Introduction to astigmatism and corneal irregularities. Customized laser vision correction.

[10] Queirós A, Villa-Collar C, Jorge J, Gutiérrez ÁR, González-Méijome JM (2012). Multi-aspheric description of the myopic cornea after different refractive treatments and its correlation with corneal higher order aberrations. J Optom.

[11] Manns F, Ho A, Parel JM, Culbertson W (2002). Ablation profiles for wavefront-guided correction of myopia and primary spherical aberration. J Cataract Refract Surg.

[12] Chan JW, Edwards MH, Woo GC, Woo VC (2002). Contrast sensitivity after laser in situ keratomileusis one-year follow-up. J Cataract Refract Surg.

[13] Sharma M, Wachler BS, Chan CC (2007). Higher order aberrations and relative risk of symptoms after LASIK. J Refract Surg.

[14] Smadja D, Reggiani-Mello G, Santhiago MR, Krueger RR (2012). Wavefront ablation profiles in refractive surgery: description, results, and limitations. J Refract Surg.

[15] Mrochen M, Seiler T (2001). Influence of corneal curvature on calculation of ablation patterns used in photorefractive laser surgery. J Refract Surg.

[16] Stojanovic A, Wang L, Jankov MR, Nitter TA, Wang Q (2008). Wavefront optimized versus custom-Q treatments in surface ablation for myopic astigmatism with the WaveLight ALLEGRETTO laser. J Refract Surg.

[17] Koller T, Iseli HP, Hafezi F, Mrochen M, Seiler T (2006). Q-factor customized ablation profile for the correction of myopic astigmatism. J Cataract Refract Surg.

[18] Gatinel D, Azar DT, Dumas L, Malet J (2014). Effect of anterior corneal surface asphericity modification on fourth-order Zernike spherical aberrations. J Refract Surg.

[19] George MR, Shah RA, Hood C, Krueger RR (2010). Transitioning to optimized correction with the WaveLight ALLEGRETTO WAVE: case distribution, visual outcomes, and wavefront aberrations. J Refract Surg.

[20] Courtin R, Saad A, Grise-Dulac A, Guilbert E, Gatinel D (2016). Changes to corneal aberrations and vision after monovision in patients with hyperopia after using a customized aspheric ablation profile to increase corneal asphericity (Q-factor). J Refract Surg.

[21] Mearza AA, Muhtaseb M, Aslanides IM (2008). Visual and refractive outcomes of LASIK with the SCHWIND ESIRIS and WaveLight ALLEGRETTO WAVE Eye-q excimer lasers: a prospective, contralateral study. J Refract Surg.

[22] Shetty R, Shroff R, Deshpande K, Gowda R, Lahane S, Jayadev C (2017). A prospective study to compare visual outcomes between wavefront-optimized and topography-guided ablation profiles in contralateral eyes with myopia. J Refract Surg.

[23] Roe JR, Manche EE (2019). Prospective, randomized, contralateral eye comparison of wavefront-guided and wavefront-optimized laser in situ keratomileusis. Am J Ophthalmol.

[24] Alves EM, Lyra AF, Tenório M, Mesquita N, Bacelar C, Montenegro A (2022). Femtosecond laser-assisted in situ keratomileusis with topography-guided or asphericity-adjusted derived data: a comparative contralateral eye study. BMC Ophthalmol.

[25] Santhiago MR, Smadja D, Gomes BF, Mello GR, Monteiro ML, Wilson SE (2014). Association between the percent tissue altered and post-laser in situ keratomileusis ectasia in eyes with normal preoperative topography. Am J Ophthalmol.

[26] Tawfik A, Eid AM, Hasanen R, Moftah IA (2014). Q-value customized ablation (custom-Q) versus wavefront optimized ablation for primary myopia and myopic astigmatism. Int Ophthalmol.

[27] Mai ELC, Lian I, Chih-Cheng L, Chang C (2018). Custom-Q vs. wavefront optimized lasik ablation treatment profile in high myopic Asian eyes. SM Opthalmol J..

[28] Gad RE, Hosny M, Ahmed RA, Sherif AM, Salah EY (2021). Contralateral eye study of topography guided versus Q value adjusted photorefractive keratectomy in myopia and myopic astigmatism. Clin Ophthalmol.

[29] Hou J, Wang Y, Lei Y, Zheng X (2018). Comparison of effective optical zone after small-incision lenticule extraction and femtosecond laser-assisted laser in situ keratomileusis for myopia. J Cataract Refract Surg.

[30] Ryan A, O’Keefe M (2012). Wavefront-guided and aspheric ablation for myopia—one-year results of the zyoptix personalized treatment advanced algorithm. Am J Ophthalmol.

[31] Sia RK, Ryan DS, Stutzman RD, Pasternak JF, Eaddy JB, Logan LA (2015). Wavefront guided versus wavefront-optimized photorefractive keratectomy: clinical outcomes and patient satisfaction. J Cataract Refract Surg.

[32] Faramarzi A, Moshirfar M, Karimian F, Delfazayebaher S, Kheiri B (2017). Aspheric versus wavefront-guided aspheric photorefractive keratectomy in eyes with significant astigmatism. J Cataract Refract Surg.

